# Droplet digital polymerase chain reaction (ddPCR) for the detection of *Plasmodium knowlesi* and *Plasmodium vivax*

**DOI:** 10.1186/s12936-020-03314-5

**Published:** 2020-07-10

**Authors:** Punitha Mahendran, Jonathan Wee Kent Liew, Amirah Amir, Xiao-Teng Ching, Yee-Ling Lau

**Affiliations:** 1 Department of Biomedical Science, Faculty of Medicine, University of Malaya, 50603 Kuala Lumpur, Malaysia; 2 Department of Parasitology, Faculty of Medicine, University of Malaya, 50603 Kuala Lumpur, Malaysia; 3 Canvio Sdn. Bhd, Setia Alam, 40170 Shah Alam, Selangor Malaysia

**Keywords:** Droplet digital polymerase chain reaction, *Plasmodium knowlesi*, *Plasmodium vivax*, Malaria

## Abstract

**Background:**

*Plasmodium knowlesi* and *Plasmodium vivax* are the predominant *Plasmodium* species that cause malaria in Malaysia and play a role in asymptomatic malaria disease transmission in Malaysia. The diagnostic tools available to diagnose malaria, such as microscopy and rapid diagnostic test (RDT), are less sensitive at detecting lower parasite density. Droplet digital polymerase chain reaction (ddPCR), which has been shown to have higher sensitivity at diagnosing malaria, allows direct quantification without the need for a standard curve. The aim of this study is to develop and use a duplex ddPCR assay for the detection of *P. knowlesi* and *P. vivax*, and compare this method to nested PCR and qPCR.

**Methods:**

The concordance rate, sensitivity and specificity of the duplex ddPCR assay were determined and compared to nested PCR and duplex qPCR.

**Results:**

The duplex ddPCR assay had higher analytical sensitivity (*P. vivax* = 10 copies/µL and *P. knowlesi* = 0.01 copies/µL) compared to qPCR (*P. vivax* = 100 copies/µL and *P. knowlesi* = 10 copies/µL). Moreover, the ddPCR assay had acceptable clinical sensitivity (*P. vivax *= 80% and *P. knowlesi* = 90%) and clinical specificity (*P. vivax* = 87.84% and *P. knowlesi* = 81.08%) when compared to nested PCR. Both ddPCR and qPCR detected more double infections in the samples.

**Conclusions:**

Overall, the ddPCR assay demonstrated acceptable efficiency in detection of *P*. *knowlesi* and *P*. *vivax*, and was more sensitive than nested PCR in detecting mixed infections. However, the duplex ddPCR assay still needs optimization to improve the assay’s clinical sensitivity and specificity.

## Background

Malaria is one of the most significant parasitic diseases that is responsible for high global morbidity and mortality. Approximately 228 million malaria cases were reported worldwide, causing an estimated 405,000 deaths in 2018 [1]. Human malaria is a mosquito-borne infectious disease which is caused by five parasite species of genus *Plasmodium*. *Plasmodium vivax, Plasmodium falciparum, Plasmodium ovale,* and *Plasmodium malariae* are known to cause human malaria. The fifth species, *Plasmodium knowlesi,* is a parasite originating from macaques. This zoonotic species has presently been reported to cause serious illness in humans, predominantly in Southeast Asia [1, 2].

Malaysia aims to eliminate malaria by year 2020. However, while the cases of human-only *Plasmodium* species have fallen substantially, the incidence of zoonotic malaria caused by *P. knowlesi* continues to increase, presenting a major challenge to regional control efforts [1, 2]. This is further complicated by a few reports of zoonotic malaria by another simian malaria parasite, i.e., *Plasmodium cynomolgi* in Malaysia [3–5]. Nonetheless, throughout 2013–2017, both *P. knowlesi* and *P. vivax* have been the predominant species causing malaria in Malaysia [6]. Furthermore, asymptomatic and sub-microscopic knowlesi and vivax malaria potentially act as silent reservoir and can contribute to disease transmission [5, 7–9].

Microscopy, a gold standard diagnostic method for malaria, faces difficulties in distinguishing *P. knowlesi* from *P. falciparum* and *P. malariae* because of their morphological similarities. *Plasmodium knowlesi* resembles *P. falciparum* in its early trophozoite stage as they both can have double chromatin dots, appliqué form and multiple infections of erythrocytes, whereas, the later erythrocytic stages of *P. knowlesi* resemble *P. malariae* with elongated trophozoites (band-form) [10].

Nested PCR and qPCR are sensitive molecular methods used widely for *Plasmodium* species detection [11–15]. However, nested PCR does not provide quantification of the parasite density. Although qPCR can detect and quantify malaria parasites, it is a challenging process as a standard curve must be generated and it is difficult to compare qPCR results across laboratories without the reference standard curve. The analytical sensitivity of these molecular assays is about 100–1000 parasites/mL depending on the blood volume [13, 14]. The droplet digital polymerase chain reaction (ddPCR) is a novel technology that provides absolute and direct quantification of target DNA [16]. ddPCR may yield more accurate results than qPCR and the results obtained from ddPCR can be compared directly across laboratories without the need for a standard curve [17]. ddPCR assay has been shown to provide high sensitivity when used to diagnose four human *Plasmodium* species where the lowest level of detection was 11 parasites/mL of blood for *Plasmodium* genus [18]. However, these assays did not include *P. knowlesi* [18, 19]. The specific aim of this study is to use a duplex ddPCR assay for the detection of *P. knowlesi* and *P. vivax*, suitable to be used in the Malaysian context, and to compare the results of this assay to those of nested PCR and qPCR.

## Methods

### Samples

Dried blood spots (DBS) from microscopically diagnosed *P. vivax* or *P. knowlesi* patients and malaria microscopy-negative thin blood smears were obtained from Sarawak and Sabah, respectively. These samples were obtained from patients where microscopy had been performed on their blood films by the admitting hospital and further verified by experienced microscopists at the district/state level. Blood samples taken from 17 healthy individuals with no history of malaria infection were used as negative controls in this study. The presence of malaria parasites in these specimens was first determined using nested PCR described below. A total of 114 samples from six groups: (i) *P. knowlesi* (40 DBS samples); (ii) *P. vivax* (40 DBS samples); (iii) healthy donors (17 DBS samples); (iv) microscopy-negative (12 blood smear samples); (v) other *Plasmodium* species: *P. malariae* (1 DNA sample), *P. falciparum* (1 DBS sample) and *P. ovale* (1 DBS sample); and, vi) non-malaria parasitic infections: *Toxoplasma gondii* (1 DNA sample) and *Dirofilaria immitis* (1 DNA sample) were used in this study. Approval for the use of these samples was obtained from the University of Malaya Medical Centre Ethics Committee (Reference no: 817.18).

### DNA extraction from DBS and thin blood smears

DNA was extracted from DBS and blood smears using QIAGEN DNeasy Blood and Tissue Kit (QIAGEN, Hilden, Germany) following the manufacturer’s protocol. One dried blood spot, approximately 1 cm in diameter, collected on qualitative filter paper (No. 101) was cut into strips and placed in a 1.5-mL centrifuge tube using sterile forceps. Then, 180 μL of buffer ATL was added, followed by the addition of Proteinase K and incubation at 56 °C for 1 h. For DNA extraction from thin blood smears, 50 μL of buffer ATL was pipetted onto the thin blood film and the smear was scraped using coverslip in a circular manner. The smear was transferred into a 1.5-mL centrifuge tube and 130 μL of buffer ATL was added. This was then followed by the addition of Proteinase K and incubation at 56 °C for 1 h. Then, the procedure that follows was according to that of the manufacturer’s protocol. Purified DNA was eluted from the column with 30 μL elution buffer and this DNA was stored at -20 °C until further use.

### Nested PCR assay

All samples were first screened and confirmed via nested PCR assay targeting the *Plasmodium* small subunit ribosomal RNA (ssRNA), as described [15, 20] before proceeding with ddPCR. The *T. gondii* and *D. immitis*-positive DNA samples were used to check for cross-reactivity of the assays. Four microlitres of DNA sample were used for the initial PCR reaction. Nest 1 amplification was performed with a preliminary 5-min denaturation at 94 °C, followed by 35 cycles of 30 s at 94 °C, 1 min at 58 °C, and 1 min at 72 °C, with a final 8-min extension at 72 °C. The nest 2 amplification was performed similarly, except 4 μL of PCR product from nest 1 PCR reaction was used as template, and a 30-cycle PCR reaction with a final 5-min extension at 72 °C were used. The amplified products were visualized through gel electrophoresis using 2% agarose gel stained with SYBR Safe DNA gel stain.

### Generation of non-linearized plasmid DNAs

Primers for *P. vivax apical membrane antigen*-*1* (*AMA*-*1*) gene (Table 1) and *P. knowlesi plasmepsin* gene (Table 1) were used for amplification of PCR fragments from both *P. vivax* and *P. knowlesi* genomic DNA. The PCR fragments were then cloned into pGEM^®^-T vectors (Promega, Madison, USA) and transformed into *Escherichia coli* TOP 10F’ cells (Invitrogen, Carlsbad, USA). Positive recombinant clones were selected by colony PCR using M13 universal primers. Purified plasmid DNA was measured using NanoQuant Plate™ (TECAN. Mannedorf, Switzerland) following the manufacturer’s instructions. Both plasmid DNA samples of *P. vivax AMA*-*1* and *P. knowlesi plasmepsin* were required to have OD 260/280 ratio of between 1.8 and 2.0. The copy number of plasmids was calculated using the following equation:

$$({\text{X g}}/\mu {\text{L DNA}}/[{\text{nucleotide transcript length}}\, \times \, 6 60])\, \times \, 6.0 2 2\, \times \, 10^{ 2 3} \, = \,{\text{Y DNA copies}}/{{\mu L}}$$

**Table 1:** List of primers and probes targeting *AMA*-*1* gene for *Plasmodium vivax* and *plasmepsin* gene for *Plasmodium knowlesi*

Species	Primer or probe (amplicon length [bp])	Sequence (5′–3′)
*P. vivax*	Primer, forward Primer, reverse HEX Probe (150)	ACGCCAAGTTCGGATTATGG CCGTCATTTCTTCTTCATACTGAG HEX-TTGATCTGAGGCACTCGCTCCG-BHQ1
*P. knowlesi*	Primer, forward Primer, reverse FAM Probe (118)	TAACATGGTAATCATACATAAGG TAAGGAAATGCCAACTCTTG 6-FAM-TCAGCCAACAACACTTACAG-BHQ1

Each non-linearized plasmid DNA was serially diluted and used in subsequent experiments for detection in ddPCR and qPCR.

### Droplet digital PCR assay for *Plasmodium* species detection

The duplex ddPCR assay targets *AMA*-*1* gene of *P.* *vivax* and *plasmepsin* gene of *P*. *knowlesi*. The duplex ddPCR reaction was prepared in a total volume of 20 μL per reaction with 1 μL of DNA sample. Probes and primers sequences used were described previously [14], with some modifications to the fluorescent dyes of the probes, i.e., HEX fluorescent dye on the *P. vivax AMA*-*1* probe and 6-FAM fluorescent dye on the *P. knowlesi plasmepsin* probe (Table 1). The ddPCR reaction mixtures were loaded to a Bio-Rad QX200 Droplet generator for generation of 12,000–20,000 droplets. Droplets were transferred to a PCR plate and standard PCR was performed using a Bio-Rad Thermal Cycler. The conventional PCR was run at 95 °C for 10 min, 40 cycles of 94 °C for 30 s and 55 °C for 1 min, and 98 °C for 10 min. After PCR, the droplets were analysed by the Bio-Rad QX200 Droplet Reader. Data analysis was then performed using Bio-Rad QuantaSoft software whereby the threshold was set manually across the entire reaction plate to separate positive and negative clusters based on the no-template control. This provided the number of positive and negative droplets, as well as quantification of *P. vivax AMA*-*1* gene and *P. knowlesi plasmepsin* gene, expressed as copies/μL in each ddPCR reaction. Non-linearized plasmids containing *P. vivax AMA*-*1* gene fragment (0.01–1000 copies/μL) and *P. knowlesi plasmepsin* gene fragment (0.01–100 copies/μL) were also used as positive controls. Each sample was analysed in duplicates and quadruplicates for the diluted plasmid samples. At least two positive droplets indicated a positive test result in the ddPCR assay. The same operators performed the assay, whereby the samples were run by batches, once for each batch.

### qPCR

To compare the analytical sensitivity of ddPCR and qPCR assay as the reference method, a duplex qPCR assay was performed with the primers and probes used in ddPCR (Table 1). Serially diluted plasmids of *P. vivax AMA*-*1* and *P. knowlesi plasmepsin* (0.01–1000 copies/μL) were used as standards for calibration. The duplex qPCR assay consists a 20 μL reaction containing 1 μL of DNA sample, 0.9 µM of each primer and 0.25 µM of each probe. The qPCR amplification was performed in the Bio-Rad CFX96 real-time PCR detection system, using the following thermal cycling condition: initial denaturation at 95 °C for 3 min followed by 40 cycles of denaturation at 94 °C for 30 s and annealing/extension at 55 °C for 1 min. All samples and non-linearized plasmid standards of both species were run in duplicate wells. Results were interpreted as positive when the Cq value was lower than 38.5.

### Data analysis

The sensitivity and specificity of ddPCR assay for *P. vivax AMA*-*1* and *P. knowlesi Plasmepsin* were calculated using nested PCR as the reference. Sensitivity and specificity (%) were calculated as follows:

$$\% {\text{ Sensitivity}} = \frac{\text{Number of true positives}}{{{\text{Number of true positives }} + {\text{number of false negatives}}}} \times 100$$

$$\% {\text{ Specificity}} = \frac{\text{Number of true negatives}}{{{\text{Number of true negatives }} + {\text{number of false positives}}}} \times 100$$

## Results

### Droplet digital PCR assay for *Plasmodium vivax* and *Plasmodium knowlesi* detection

**Fig. 1:**
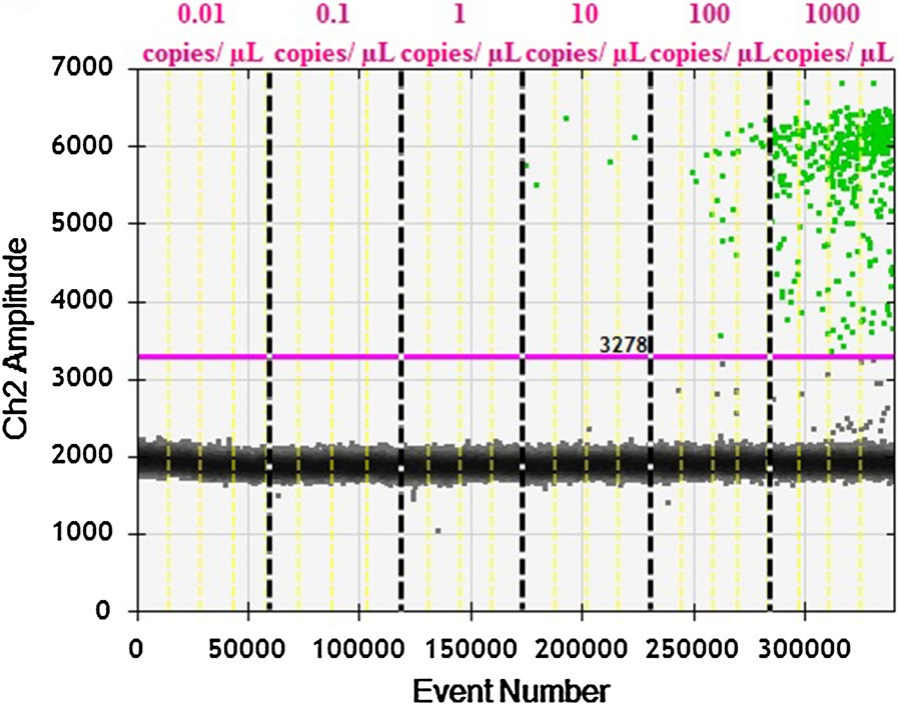
The one-dimensional (1D) ddPCR results of *Plasmodium vivax AMA*-*1* gene detection assay. ddPCR sample wells is numbered in pink according to plasmid dilution contents. The pink lines represent the manually-assigned threshold where the threshold for positive detection was 3278 relative fluorescence unit (rfu). The yellow vertical dotted line separate results of individual reaction well

**Fig. 2:**
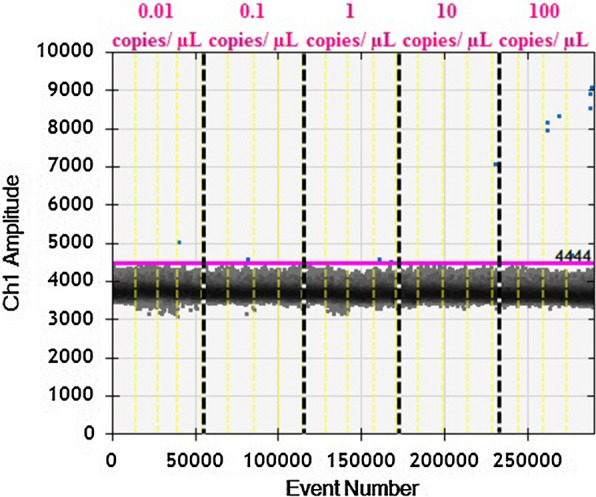
The one-dimensional (1D) ddPCR results of *Plasmodium knowlesi Plasmepsin* gene detection assay. ddPCR sample wells is numbered in pink according to plasmid dilution contents. The pink lines represent the manually assigned threshold where the threshold for positive detection was 4444 relative fluorescence unit (rfu). The yellow vertical dotted line separate results of individual reaction well

The one-dimensional (1D) ddPCR results from Bio-Rad QX100TM Droplet Reader of *P. vivax AMA*-*1* gene and *P. knowlesi plasmepsin* gene are shown in Fig. 1 and Fig. 2. The threshold for positive detection was 3278 relative fluorescence unit (rfu) for *P. vivax* and 4444 rfu for *P. knowlesi*.

### Evaluation of clinical sensitivity and clinical specificity of ddPCR assay for the detection of *Plasmodium vivax* and *Plasmodium knowlesi*

The analysis of the 114 clinical samples screened using ddPCR compared to nested PCR is shown in Table 2. Concordance rate between the two assays were 69.30%. The highest disagreement between the assays occurred among the healthy donor samples. The calculated values for sensitivity and specificity of ddPCR for the detection of *P. vivax AMA*-*1* were 80% (32/40) and 87.84% (65/74), respectively. Sensitivity and specificity of *P. knowlesi Plasmepsin* in the ddPCR assay were found to be 90% (36/40) and 81.08% (60/74), respectively.

**Table 2:** Analysis of clinical samples using ddPCR compared to nested PCR

Sample	ddPCR (number of samples)				Number of samples
	Positive			Negative	
	*P. vivax*	*P. knowlesi*	Mixed (*P. vivax* and *P. knowlesi*)		
Nested PCR-confirmed *P. vivax*	28	0	4	8	40
Nested PCR-confirmed *P. knowlesi*	0	28	8	4	40
*P. malariae*	0	0	0	1	1
*P. falciparum*	0	0	0	1	1
*P. ovale*	0	0	0	1	1
*T. gondii*	0	0	0	1	1
*D. immitis*	0	0	0	1	1
Healthy donors	0	9	0	8	17
Microscopy-negative	1	1	0	10	12

### Comparison between nested PCR, ddPCR and qPCR assays

Due to limited samples, only 30 *P. vivax* samples, 29 *P. knowlesi* samples, 1 *P. ovale* sample, 1 *P. falciparum* sample, and 12 microscopy-negative samples (total = 73 nested PCR-confirmed samples) were available for comparison between the 3 assays, i.e., nested PCR, ddPCR and qPCR.

Results of the 3 PCR assays are shown in Table 3. For these 73 samples tested, concordance rate between the 3 assays were 75.34%. Both qPCR and ddPCR detected double infections among the nested PCR confirmed-*P. vivax* or *P. knowlesi* mono-infected samples. While ddPCR failed to detect *Plasmodium* parasites in 7 of the positive samples, both ddPCR and qPCR did manage to detect presence of *Plasmodium* in 2–3 of the microscopy-negative samples. Although both ddPCR and qPCR assays produced comparable overall results, qPCR was more sensitive at detection compared to ddPCR, identifying slightly more double infections and positive samples than ddPCR.

**Table 3:** Comparison of results between nested PCR, ddPCR and qPCR

Samples	Total	ddPCR result				qPCR result			
		Pv	Pk	Pv + Pk	Negative	Pv	Pk	Pv + Pk	Negative
Nested PCR-confirmed Pv only	30	24	0	2	4	23	0	6	1
Nested PCR-confirmed Pk only	29	0	21	5	3	1	21	7	0
Other *Plasmodium* spp.	2	0	0	0	2	0	0	0	2
Microscopy - negative	12	1	1	0	10	3	0	0	9
Total	73	25	22	7	19	27	21	13	12

Pv, *P. vivax*; Pk, *P. knowlesi*

With further investigation, the number and type of samples for which the results from the ddPCR and qPCR assays were in agreement or discordance are shown in Table 4. The concordance between the two assays was 65.75%. The results in Table 4, further corroborated the overall finding that qPCR identified slightly more double infections and positive samples than ddPCR.

**Table 4:** Number and type of samples in agreement or discordance based on results from ddPCR and qPCR assays

Agreement	Pv	Pk	Pv + Pk	Negative
Nested PCR-confirmed Pv only (n = 30)	19	–	–	1
Nested PCR-confirmed Pk only (n = 29)	–	15	1	–
Other *Plasmodium* spp. (n = 2)	–	–	–	2
Microscopy–negative (n = 12)	1	–	–	9
**Discordance**	**qPCR result – ddPCR result**			
	Pv (qPCR) − Pv + Pk (ddPCR)	Pv + Pk (qPCR) − Pv (ddPCR)	Pv (qPCR) - negative (ddPCR)	Pv + Pk (qPCR) − negative (ddPCR)
Nested PCR-confirmed Pv only (n = 30)	2	5	2	1
	Pk (qPCR) − Pv + Pk (ddPCR)	Pv (qPCR) − Pv + Pk (ddPCR)	Pv + Pk (qPCR) − Pk (ddPCR)	Pk (qPCR) − negative (ddPCR)
Nested PCR-confirmed Pk only (n = 29)	3	1	6	3
	Pv (qPCR) − Pk (ddPCR)	Pv (qPCR) − negative (ddPCR)		
Microscopy-negative (n = 12)	1	1		

Pv, *P. vivax*; Pk, *P. knowlesi*

### Comparison of analytical sensitivity between ddPCR and qPCR assays

**Fig. 3:**
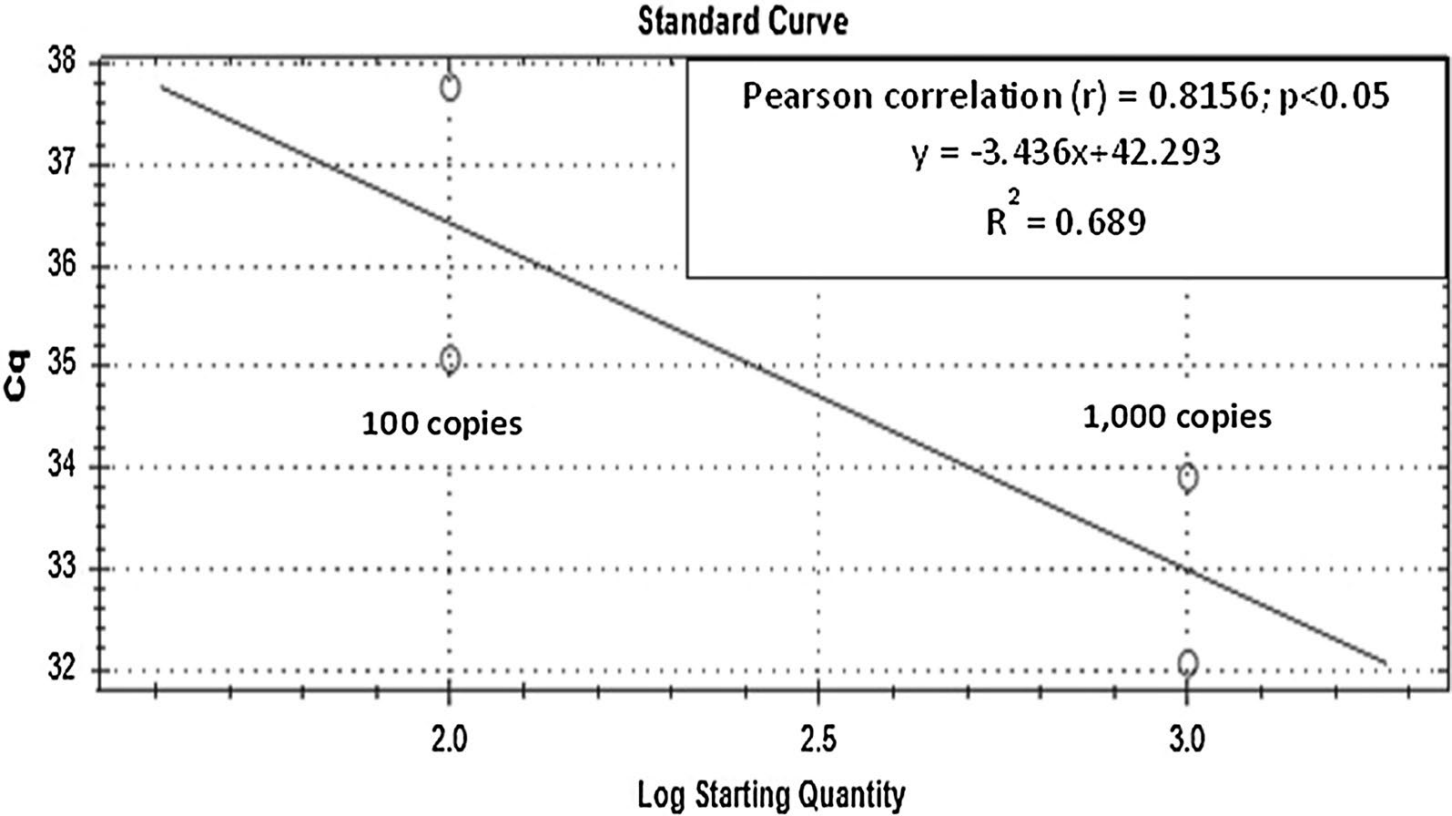
Standard curve of qPCR assay run with positive plasmid DNA of *Plasmodium vivax AMA*-*1.* Plasmid DNA was ten-fold diluted serially from 1000 to 0.01 copies/μL. The slope of the plasmid DNA standard curve is –3.436, equivalent to an efficiency of 95.5.7% (*R*^2^ = 0.689)

**Fig. 4:**
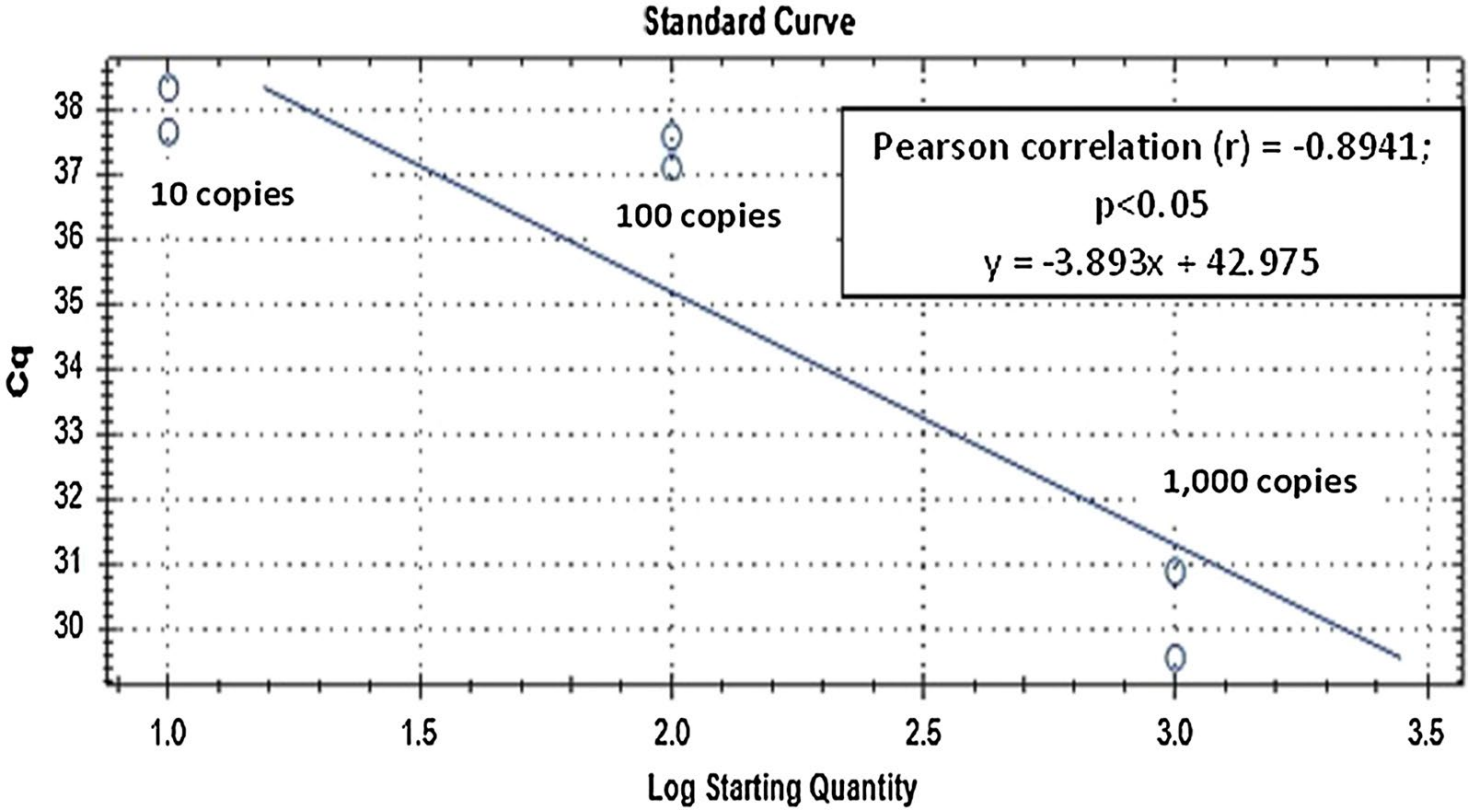
Standard curve of qPCR assay run with positive plasmid DNA of *Plasmodium knowlesi Plasmepsin.* Plasmid DNA was ten-fold diluted serially from 1000 to 0.01 copies/μL. The slope of the plasmid DNA standard curve is − 3.893, equivalent to an efficiency of 80.7% (*R*^2^ = 0.799)

**Fig. 5:**
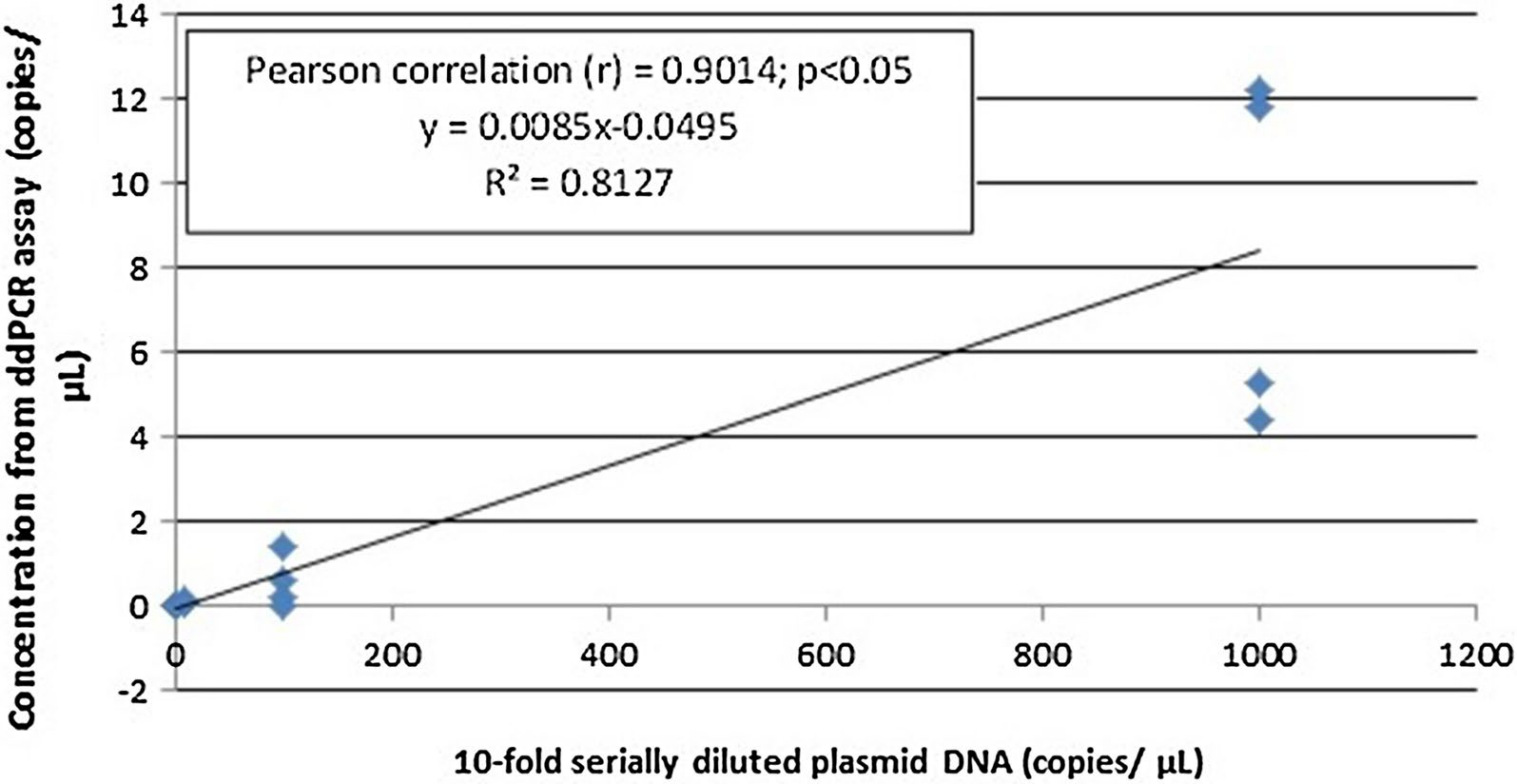
Linear regression of the ddPCR assay for positive plasmid DNA of *Plasmodium vivax AMA*-*1* (1000–0.01 copies/µL). The estimated Pearson correlation coefficient of the plasmid DNA regression curve (y = 0.0085x – 0.0495) is 0.9014 (*R*^2^ = 0.8127, *P *< 0.05)

**Fig. 6:**
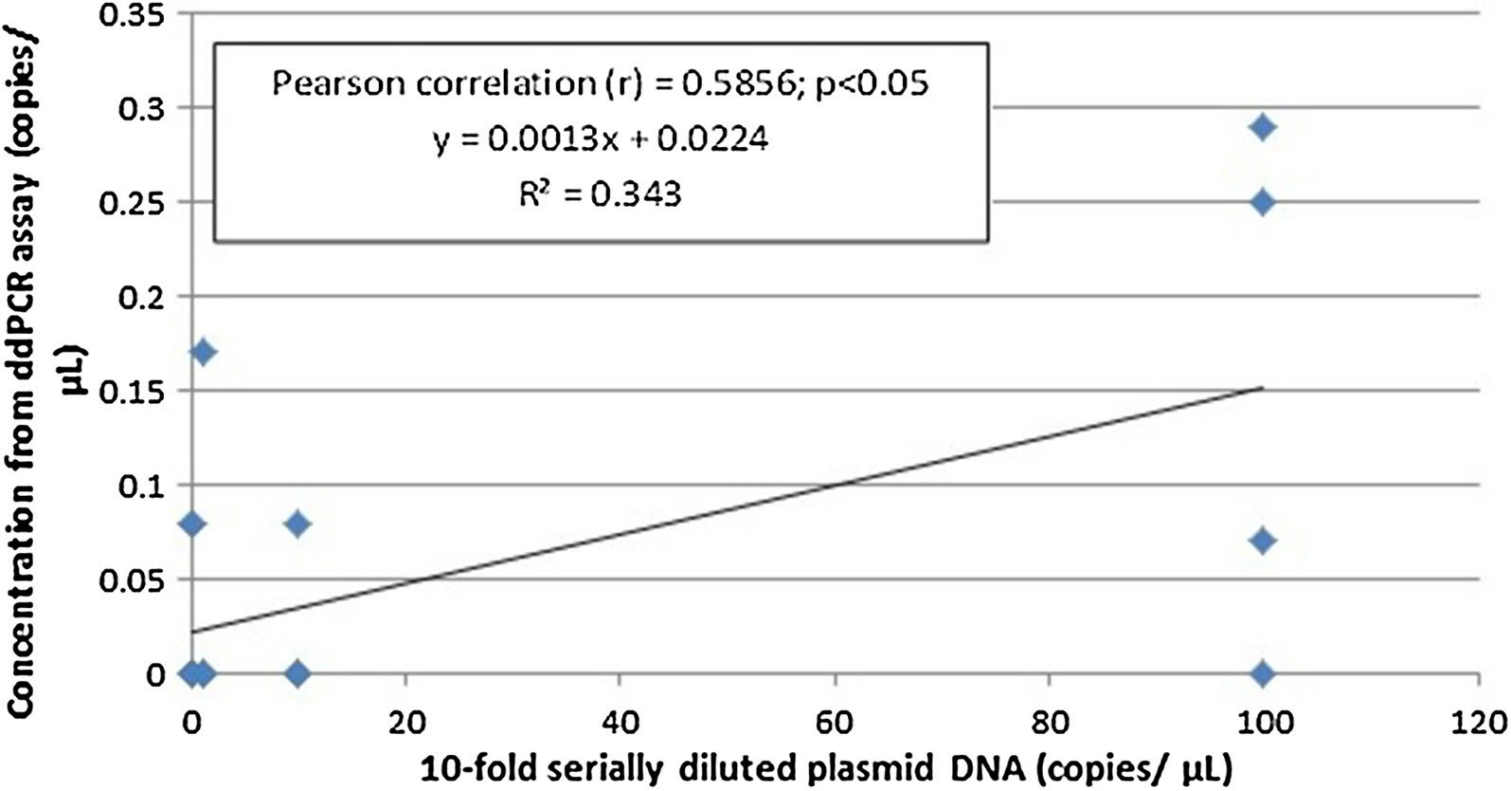
Linear regression of the ddPCR assay for positive plasmid DNA of *Plasmodium knowlesi Plasmepsin* (100–0.01 copies/µL). The estimated Pearson correlation coefficient of the plasmid DNA regression curve (y = 0.0013x + 0.0224) is 0.5856 (*R*^2^ = 0.343, *P *< 0.05)

The standard curves for the qPCR assay and the linear regression curve for the ddPCR assay to compare the analytical sensitivity of both assays were constructed by using ten-fold serially diluted non-linearized plasmid DNA of *P. vivax AMA*-*1* gene and *P. knowlesi plasmepsin* gene. The qPCR assay for the detection of *P. vivax AMA*-*1* (Fig. 3) exhibited linearity (R2 = 0.689, P < 0.05) with the dynamic range tested using the plasmid DNA (1000–0.01 copies/μL). In the qPCR standard curve for *P. vivax*, the slope was − 3.436 for the positive plasmid DNA, equivalent to a PCR efficiency of 95.5%. According to the standard curves, the limit of sensitivity of the qPCR test for plasmid DNA of *P. vivax AMA*-*1* is 100 copies/μL. As for *P. knowlesi plasmepsin*, the qPCR assay (Fig. 4) exhibited linearity (R^2^ = 0.799, P < 0.05) with the dynamic range tested using the plasmid DNA (1000–0.01 copies/μL). In the qPCR standard curve for *P. knowlesi*, the slope was − 3.893 for the positive plasmid DNA, equivalent to a PCR efficiency of 80.7%. According to the standard curve, the sensitivity of the qPCR test for non-linearized plasmid DNA of *P. knowlesi Plasmepsin* is 10 copies/μL. Apart from that, by quantifying plasmid DNA standards, the linearity of ddPCR assay was also determined. The measurements of ddPCR assay showed positive plasmid DNA standards of *P. vivax AMA*-*1* (Fig. 5) exhibited linearity (R2 = 0.8127, P < 0.05) over the measured dynamic range (1000–0.01 copies/μL) with the slope value 0.0085. In this study, the analytical sensitivity of the ddPCR assay for plasmid DNA of *P. vivax AMA*-*1* was 10 copies/μL, which is more sensitive compared to qPCR (Fig. 3) where the analytical sensitivity was 100 copies/µL. As for *P. knowlesi plasmepsin* (Fig. 6), ddPCR assay exhibited linearity (R^2^ = 0.343, P < 0.05) over the measured dynamic range (100–0.01 copies/μL) with the slope value 0.0013. In this study, the analytical sensitivity of the ddPCR assay for non-linearized plasmid DNA of *P. knowlesi plasmepsin* was 0.01 copies/μL, which is more sensitive compared to qPCR (Fig. 4) where the lower limit of detection was 10 copies/µL.

## Discussion

The aim of this study is to use a duplex ddPCR assay to detect *P. vivax* and *P. knowlesi* at species level and compare the results to those of nested PCR and qPCR. This is the first report of *Plasmodium knowlesi* detection using ddPCR. This method is able to detect *P*. *knowlesi* along with *P. vivax* as they are the two most predominant species causing human malaria in Malaysia. Each species contain one copy of the gene target (*AMA*-*1* gene of *P. vivax* and *plasmepsin* gene of *P. knowlesi*) per parasite genome [14].

Based on standard curve of the ddPCR assay for positive plasmid DNA of *P. vivax AMA*-*1,* the assay showed higher analytical sensitivity with detection limit of 10 copies/µL (Fig. 5), than qPCR with detection limit of 100 copies (parasites)/µL (Fig. 3) which is similar to the previous report [14] where the analytical sensitivity by qPCR was 10–100 copies/µL. Similarly, ddPCR assay for positive non-linearized plasmid DNA of *P. knowlesi Plasmepsin,* showed higher sensitivity with detection limit at 0.01 copy/µL (Fig. 6), than qPCR with detection limit at 10 copies/µL (Fig. 4). However, it should be noted that in qPCR, when circular (super-coiled) plasmid standards are used, amplification products can be detected 2–4 cycles later than the corresponding linearized plasmid standards. Consequently, qPCR quantifications using non-linearized plasmid standards can be overestimated as compared to qPCR using linearized plasmid standards and compared to absolute quantification by ddPCR [19]. Nonetheless, analytical sensitivities of the ddPCR and qPCR assays in this study were assessed by using the same circular plasmids as standards. Therefore, making the results comparable between assays.

This study using duplex ddPCR assay to detect *P. vivax* and *P. knowlesi* showed acceptable clinical sensitivity (80% for *P. vivax* and 90% for *P. knowlesi*) and clinical specificity (87.84% for *P. vivax* and 81.08% for *P. knowlesi*) compared to nested PCR. The clinical sensitivity reflects the ability of the assay to correctly identify those patients with the disease while clinical specificity reflects the ability of the assay to correctly identify those patients without the disease [21]. A high clinical sensitivity of ddPCR assay is important as the assay have less chance of misdiagnosing those who have malaria. Hence, more people infected with malaria can be treated quickly with correct treatment and this can reduce severity of the disease. On the other hand, low clinical specificity indicates more false positive results are being produced by the assay. The false positive results might be attributed to several possibilities such as cross contamination of the samples, assay specificity or sub-microscopic malaria infection, which was not detected in nested PCR previously. The highly sensitive nature of ddPCR may also magnify the problem of false positives in this duplex ddPCR assay. In malaria screening, it is not feasible to use an assay with low clinical specificity, since many people without the disease will be screened positive and potentially receive unnecessary diagnostic procedures and treatments.

Concordance rate between the ddPCR and nested PCR assays for the 114 samples were 69.30%, whereby ddPCR failed to detect *Plasmodium* parasites in 12 of the positive samples. However, when the healthy donor samples were not included, concordance rate between the 3 assays were 75.34% (for 73 samples). This was because the highest disagreement between the results of the ddPCR and nested PCR assays occurred among the healthy donor samples, citing a need for further optimization of the ddPCR assay. However, both qPCR and ddPCR detected double infections in the supposedly mono-infected samples and presence of *Plasmodium* in 2–3 of the microscopy-negative samples. Nevertheless, concordance between ddPCR and qPCR was only 65.75%. qPCR was more sensitive at detection compared to ddPCR, identifying slightly more double infections and positive samples than ddPCR, further corroborating the need for further optimization of the ddPCR assay.

The above limitations of the ddPCR assay and various discrepancies between the results of the assays need to be studied carefully. Firstly, nested PCR may turn out to be more sensitive in some cases because the nested PCR amplifies the *Plasmodium 18ssRNA* gene which has about 4 to 8 copies per parasite genome [22, 23], while the duplex ddPCR and qPCR assay amplify the *P. vivax AMA*-*1* gene and *P. knowlesi plasmepsin* gene, where each exists in 1 copy per parasite genome [14]. Furthermore, the nested PCR uses two rounds of amplification, while the latter two assays use one round of amplification. Additionally, nested PCR was used to screen all samples received in the laboratory where 4 μL of DNA per PCR reaction was routinely used, compared to 1 μL used in qPCR and ddPCR. All these factors could have led to higher yield of PCR product from nested PCR than in the other two assays. These could have led to negative results by ddPCR and qPCR for malaria-positive samples. Another reason for the discrepancies could be technical error from pipetting small volume of DNA (1 μL). This error is further exacerbated by the relatively low amount of parasites in the DBS and blood smear samples. The above error may also be the reason for the difference in results between the ddPCR and qPCR assays, besides the less-than-optimized assays. On the other hand, qPCR and ddPCR managed to detect double infections and parasites in microscopy-negative samples, which nested PCR failed to do. It has been documented that the nested PCR for amplification of *P. knowlesi 18ssRNA* gene is less sensitive than qPCR or other PCR assays targeting different genes with higher copy numbers [8, 24]. This could possibly be the same for specific amplification of *P. vivax* DNA, although this needs confirmation.

However, in this study, the duplex ddPCR assay had better analytical sensitivity than qPCR for both *P. vivax* and *P. knowlesi* at lower copy numbers. ddPCR assay yielding higher sensitivity has been reported in studies detecting the four other human *Plasmodium* species [18, 19]. Moreover, it has already been shown that the nested PCR assay has lower sensitivity at detecting asymptomatic and sub-microscopic *P. knowlesi* infections [24]. Thus, the ddPCR assay for the detection of *P. knowlesi* based on *Plasmepsin* gene potentially offers a method with high sensitivity that improves *Plasmodium* species identification and quantification. This can be utilized as a research tool to diagnose sub-patent and sub-microscopic knowlesi malaria infection reported in previous studies in Malaysia [5, 7–9]. As such, the clinical performance of this duplex ddPCR assay needs to be optimized for higher specificity and sensitivity, and further compared to that of qPCR using a larger panel of samples in the future. Technical areas for improvement include addition of more DNA template, optimization of annealing temperature and concentration of primers and probes.

Apart from the above, the ddPCR assay can be further developed into a 5-plex ddPCR assay to allow detection of all five *Plasmodium* species known to cause malaria in humans as multiplexing ddPCR assay reduces usage of resources and preparation time. This may help to make this method more cost- and time-effective as the ddPCR assay can be relatively more expensive and more time consuming. However, although the above is an ideal approach, this multiplex ddPCR assay should be customized according to regional malaria prevalence or depending on diagnostic, epidemiological or research purpose. For example, including detection of *P. ovale* or *P. malariae* in the multiplex ddPCR for diagnostic purpose is not practical in Malaysia or some Southeast Asian countries, as they are hardly seen in these regions. Nonetheless, the assay may include detection of other zoonotic simian malaria parasites such as *P. cynomolgi*. Natural infection of *P. cynomolgi* was first reported in Peninsular Malaysia [3], followed by other reports from Sabah and Sarawak [4, 5]. It is now also found naturally transmitted in Cambodia [24], and in a traveller returning to Denmark [25]. Unfortunately, the current study could not include *P. cynomolgi* for evaluation of specificity of the duplex assay due to the lack of *P. cynomolgi* mono-infected DNA sample.

Despite its limitations, the duplex ddPCR assay provides a relatively sensitive detection and quantitative method for detection of malaria parasites. By utilizing ddPCR, the data on parasite densities measured and obtained can be compared directly across laboratories. It can also be an effective tool for epidemiological studies for the detection of asymptomatic and sub-microscopic malaria infection.

## Conclusions

This study shows that the duplex ddPCR assay is potentially more sensitive in detecting *P. knowlesi* and *P. vivax* at low parasite density compared to qPCR. Hence, ddPCR can be used as a research tool for large field studies containing high proportions of low-density malaria infections as it contributes to similar, if not more sensitive results than qPCR as being supported by previous studies [18, 19]. Further optimization of this ddPCR assay is crucial to improve the assay’s clinical sensitivity and specificity in order to produce reliable and accurate results.

## Data Availability

The datasets analyzed in this study are available from the corresponding author on request.

## Abbreviations

PCR: Polymerase chain reaction
ddPCR: Digital droplet PCR
qPCR: Quantitative real-time PCR
DBS: Dried blood spots
