# Correction: Droplet digital polymerase chain reaction (ddPCR) for the detection of *Plasmodium knowlesi* and *Plasmodium vivax*

**DOI:** 10.1186/s12936-023-04733-w

**Published:** 2023-10-25

**Authors:** Punitha Mahendran, Jonathan Wee Kent Liew, Amirah Amir, Xiao-Teng Ching, Yee-Ling Lau

**Affiliations:** 1 Department of Biomedical Science, Faculty of Medicine, University of Malaya, 50603 Kuala Lumpur, Malaysia; 2 Department of Parasitology, Faculty of Medicine, University of Malaya, 50603 Kuala Lumpur, Malaysia; 3 Canvio Sdn. Bhd, Setia Alam, 40170 Shah Alam, Selangor Malaysia

**Correction: Malar J (2020) 19:241** **https://doi.org/10.1186/s12936-020-03314-5**

Following publication of the original article [1], it was brought to the journal's attention that the Funding declaration was incomplete. The declaration has since been corrected to the following:

This study was supported by the Long Term Research Grant Scheme (LRGS/1/2018/UM/01/1/4) of the Ministry of Higher Education, Malaysia.

The authors thank you for reading this erratum and apologize for any inconvenience caused.

## References

[1] Mahendran P, Liew JWK, Amir A, Ching X-T, Lau Y-L (2020). Droplet digital polymerase chain reaction (ddPCR) for the detection of *Plasmodium knowlesi* and *Plasmodium vivax*. Malar J.

